# Non-canonical chromatin-based functions for the threonine metabolic pathway

**DOI:** 10.1038/s41598-024-72394-z

**Published:** 2024-09-30

**Authors:** Jennifer K. Chik, Xue Bessie Su, Stephen Klepin, Jessica Raygoza, Lorraine Pillus

**Affiliations:** 1 Department of Molecular Biology, University of California San Diego, 9500 Gilman Drive, La Jolla, CA 92093-0347 USA; 2 Medical Research Council, Laboratory for Molecular Cell Biology, University College London, London, WC1E 6BT UK

**Keywords:** Double-strand DNA breaks, Chromatin, Eukaryote

## Abstract

The emerging class of multi-functional proteins known as moonlighters challenges the “one protein, one function” mentality by demonstrating crosstalk between biological pathways that were previously thought to be functionally discrete. Here, we present new links between amino acid metabolism and chromatin regulation, two biological pathways that are critical for cellular and organismal homeostasis. We discovered that the threonine biosynthetic pathway is required for the transcriptional silencing of ribosomal DNA (rDNA) in *Saccharomyces cerevisiae*. The enzymes in the pathway promote rDNA silencing through distinct mechanisms as a subset of silencing phenotypes was rescued with exogenous threonine. In addition, we found that a key pathway enzyme, homoserine dehydrogenase, promotes DNA repair through a mechanism involving the MRX complex, a major player in DNA double strand break repair. These data further the understanding of enzymes with non-canonical roles, here demonstrated within the threonine biosynthetic pathway, and provide insight into their roles as potential anti-fungal pharmaceutical targets.

## Introduction

Proteins have traditionally been branded by the function or activity for which they were first identified. Thus, the majority of proteins are known for a single cellular function and any additional or independent roles have generally been neither sought nor systematically investigated. To counter this limited view, recent work has highlighted the emerging class of proteins now known as *moonlighters*, which feature multiple distinct biological functions on the same polypeptide chain that cannot be attributed to gene fusion, duplication, or alternative splicing^1,2^. Importantly, moonlighting is a key biological mechanism that increases the protein coding capacity of genomes. Hundreds of moonlighters have now been documented and have been identified in each domain of life^3^. As a notable example, one of the most highly conserved proteins in biology, histone H3, was recently characterized as a copper reductase^4^. Therefore, moonlighters have the potential to expand the current understanding of established biological pathways and the functional significance of ancient proteins.

Two pathways essential to cellular and organismal wellbeing are amino acid metabolism and chromatin regulation. These pathways are inherently linked due to metabolites, such as nicotinamide adenine dinucleotide (NAD +) and S-adenosylmethionine (SAM), acting as cofactors for chromatin modifiers^5^. There is now increasing evidence for moonlighters that create additional connections between these pathways through a variety of mechanisms. Our previous work in the budding yeast *Saccharomyces cerevisiae* identified chromatin-based roles for the amino acid metabolizers homocitrate synthase and glutamate dehydrogenase in DNA damage repair and H3 N-terminal clipping and telomeric silencing, respectively^6,7^.

Beyond telomeric silencing, the ribosomal DNA (rDNA) locus is one of the three best characterized transcriptionally silenced loci in yeast. A major silencing mechanism for rDNA is regulated by Sir2, an NAD-dependent protein deacetylase that functions with Net1 and Cdc14 as part of the RENT (regulator of nucleolar silencing and telophase) complex^8^. Along with its roles in silencing, Sir2 is also critical for maintaining the genomic stability of the rDNA locus^9^. Defects in rDNA silencing can have dramatic consequences on cellular homeostasis and are associated with decreased lifespan^10–12^.

Cells are subjected daily to thousands of DNA damage events. Of the variety of DNA damage that can occur, double strand breaks (DSBs) are among the most detrimental^13^. DSBs can be caused by multiple external factors including ionizing radiation and chemotherapeutics, but are also generated by endogenous processes including meiotic recombination and replication fork collapse. If not repaired swiftly and correctly, DSBs can ultimately result in the loss of genetic information, chromosomal translocations, genomic instability, and apoptosis. There are two predominant pathways used to repair DSBs: non-homologous end joining (NHEJ) and homologous recombination. Although homologous recombination is generally favored in yeast, repair pathway choice is also dependent on other factors, including cell cycle stage. The MRX complex (MRN in other eukaryotes) is one of the “first responders” to a DSB and dictates which mechanism of repair will be used^14^. In yeast, this complex consists of Mre11, Rad50, and Xrs2, which together bridge the DSB and aid in the recruitment of repair machinery.

**Fig. 1:**
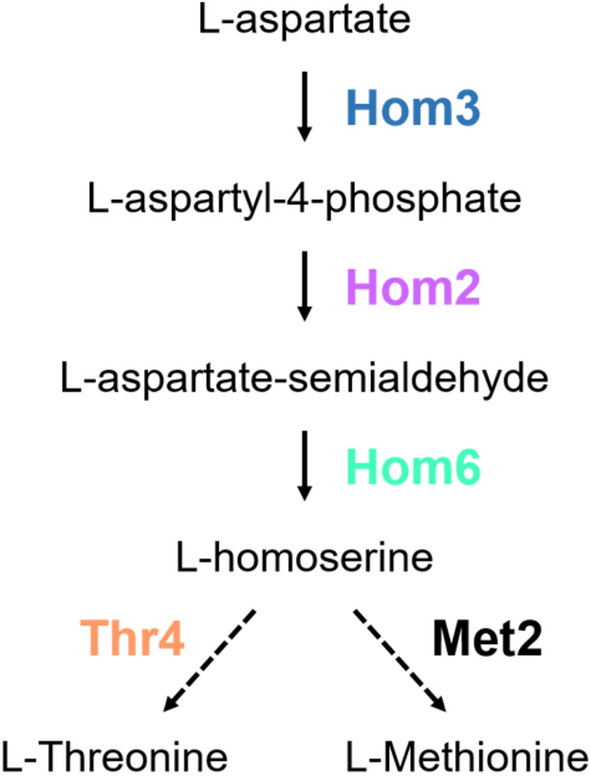
The threonine biosynthetic pathway The Superpathway of Threonine and Methionine Biosynthesis (http://tinyurl.com/ThrMetPathway).

Whereas DNA repair mechanisms are well conserved, multiple metabolic pathways, including some of those controlling amino acid synthesis, are not found in humans. Because the threonine biosynthetic pathway is found in fungi but not humans, who are auxotrophic for this essential amino acid, enzymes in the pathway stand out as attractive targets for pharmaceutical design of anti-fungal compounds^15,16^, due to the toxicity and limited clinical efficacy of currently available drugs. In yeast*,* threonine is synthesized from aspartate by sequential reactions catalyzed by a series of Hom and Thr proteins. The Hom proteins produce homoserine, the last common precursor for threonine and methionine, whereas the Thr proteins convert homoserine to threonine (Fig. 1). Homoserine dehydrogenase, Hom6, catalyzes the NAD(P)H-dependent conversion of aspartate semialdehyde to homoserine. Hom6 is an intriguing protein within this pathway as it has been well characterized structurally and has a unique active site compared to other oxidoreductases^17^. Hom6 localizes to both the cytoplasm and nucleus, yet its role in the nucleus remains largely unexplored^18^. Here, we identify previously uncharacterized roles for Hom6 and the threonine biosynthetic pathway in chromatin functions, including rDNA silencing and DSB break repair.

## Results

### rDNA silencing is regulated by the threonine biosynthetic pathway and a moonlighting function of Hom6

In a previous study, we reported an in silico screen to identify new moonlighters that act at the intersection of amino acid metabolism and chromatin regulation in *S. cerevisiae*. Notably, Hom2 and Hom6, both contributing to homoserine synthesis, were identified as potential moonlighters and were noted to have roles in rDNA silencing^7^. We therefore hypothesized that enzymes both up– and downstream in the pathway may also have roles in the regulation of silencing at the rDNA. Threonine synthase, Thr4, which acts downstream of the Hom proteins to synthesize threonine was also tagged as a moonlighting candidate in the original in silico screen, although it was eliminated in the final round of screening.

**Fig. 2:**
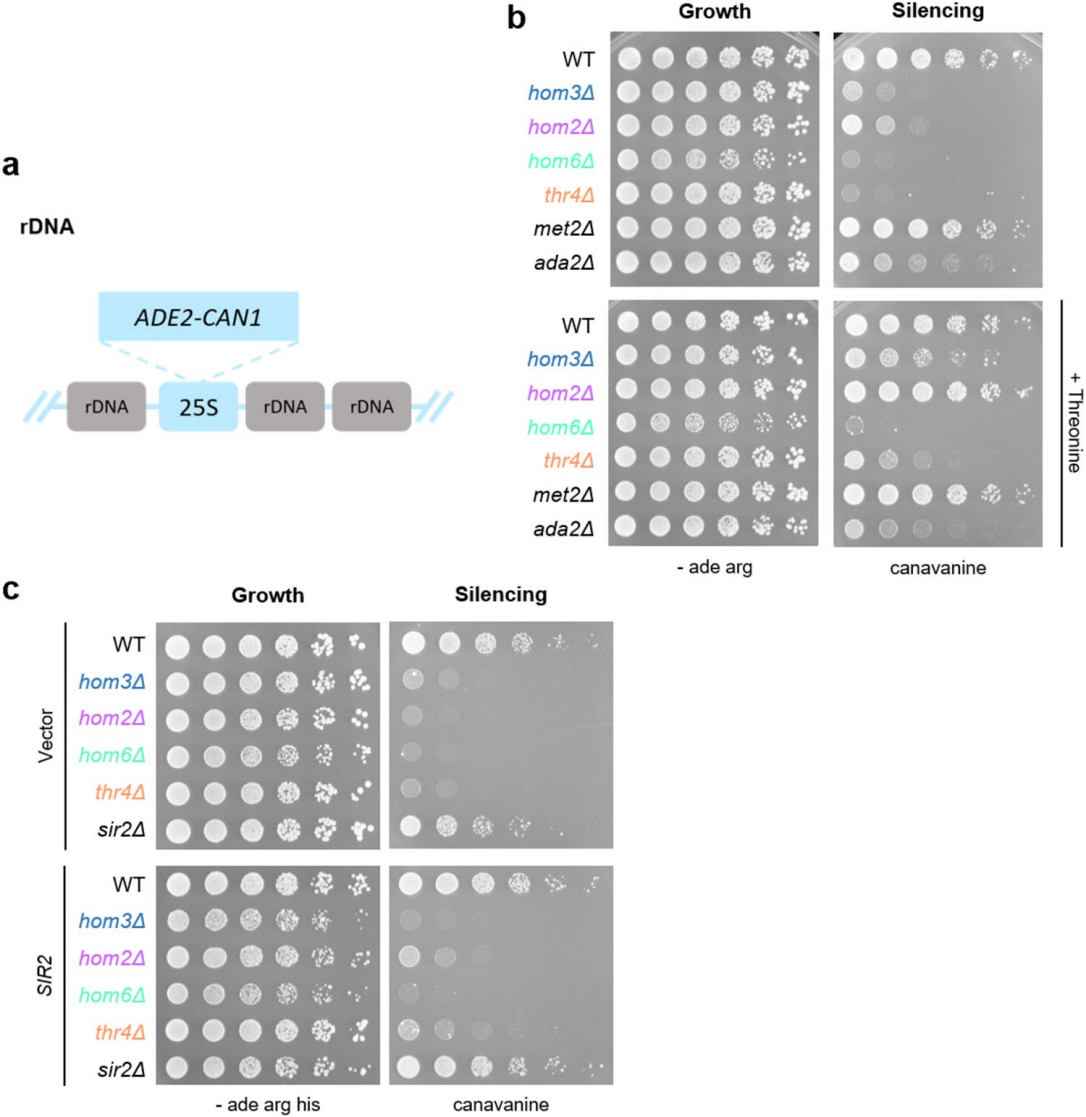
The threonine biosynthetic pathway is required for rDNA silencing. (**a**) An *ADE2-CAN1* cassette in the 25S region of the rDNA was used to monitor rDNA transcriptional silencing. Selection for growth on medium without adenine assures presence and maintenance of the cassette, whereas transcription of *CAN1* enables cellular import of canavanine. (**b**) The individual *hom3Δ, hom2Δ, hom6Δ, thr4Δ* mutants are defective in rDNA silencing on 8 µg/mL canavanine. The *hom3Δ, hom2Δ,* and *thr4Δ* silencing defects are rescued when medium is supplemented with exogenous threonine. All plates lack both adenine and arginine for marker maintenance. Imaged after a 3-day incubation. (**c**) *SIR2* overexpression does not rescue rDNA silencing defects in threonine biosynthetic pathway deletion strains. Strains were transformed with vector (pLP270) or a *SIR2* overexpression plasmid (pLP891) and assayed for silencing at the rDNA on 16 µg/mL canavanine. Imaged after a 3-day incubation. Strains shown: WT (LPY23157), *hom3Δ (*LPY16907), *hom2Δ* (LPY16020), *hom6Δ* (LPY23065,) *thr4Δ* (LPY23341), *met2Δ* (LPY23330), *ada2Δ* (LPY11674), *sir2Δ* (LPY5013).

To monitor silencing changes in the mutants, we utilized strains that have an *ADE2-CAN1* reporter inserted at the rDNA (Fig. 2a)^19,20^. The *ADE2* gene allows selection for maintenance of the reporter whereas *CAN1* serves as an indicator for silencing levels at the rDNA. When silencing is disrupted, *CAN1* is actively transcribed leading to production of the arginine permease. Canavanine, a toxic arginine analog, can then be imported into the cell, resulting in cell death. We see that in addition to *hom2Δ* and *hom6Δ* strains, other deletion strains in the threonine biosynthetic pathway are also sensitive to canavanine (Fig. 2b). These defects in rDNA silencing are specific to the threonine biosynthetic branch of the pathway as *met2Δ* strains do not exhibit growth defects when plated on canavanine (Fig. 2b).

Because *SIR2* and its gene dosage are centrally involved in rDNA silencing, we asked if rDNA silencing defects in *hom3Δ*, *hom2Δ*, *hom6Δ*, and *thr4Δ* strains could be rescued by increased *SIR2* gene dosage (Fig. 2c). Modest positive effects were only seen in *hom2Δ* and *thr4Δ* indicating that threonine biosynthetic pathway-mediated rDNA silencing is regulated by a mechanism that is not exquisitely sensitive to *SIR2* dosage.

The highly repetitive rDNA locus, on average between 150 and 200 copies, exists in a single tandem array on chromosome XII. The highly repetitive nature makes the locus subject to homologous recombination events that can lead to amplification or reduction in copy number. Silencing and stability of rDNA are closely intertwined and the resulting shifts in rDNA copy number have been shown to influence key biological processes including silencing, aging, and the DNA damage response^21–23^.

**Fig. 3:**
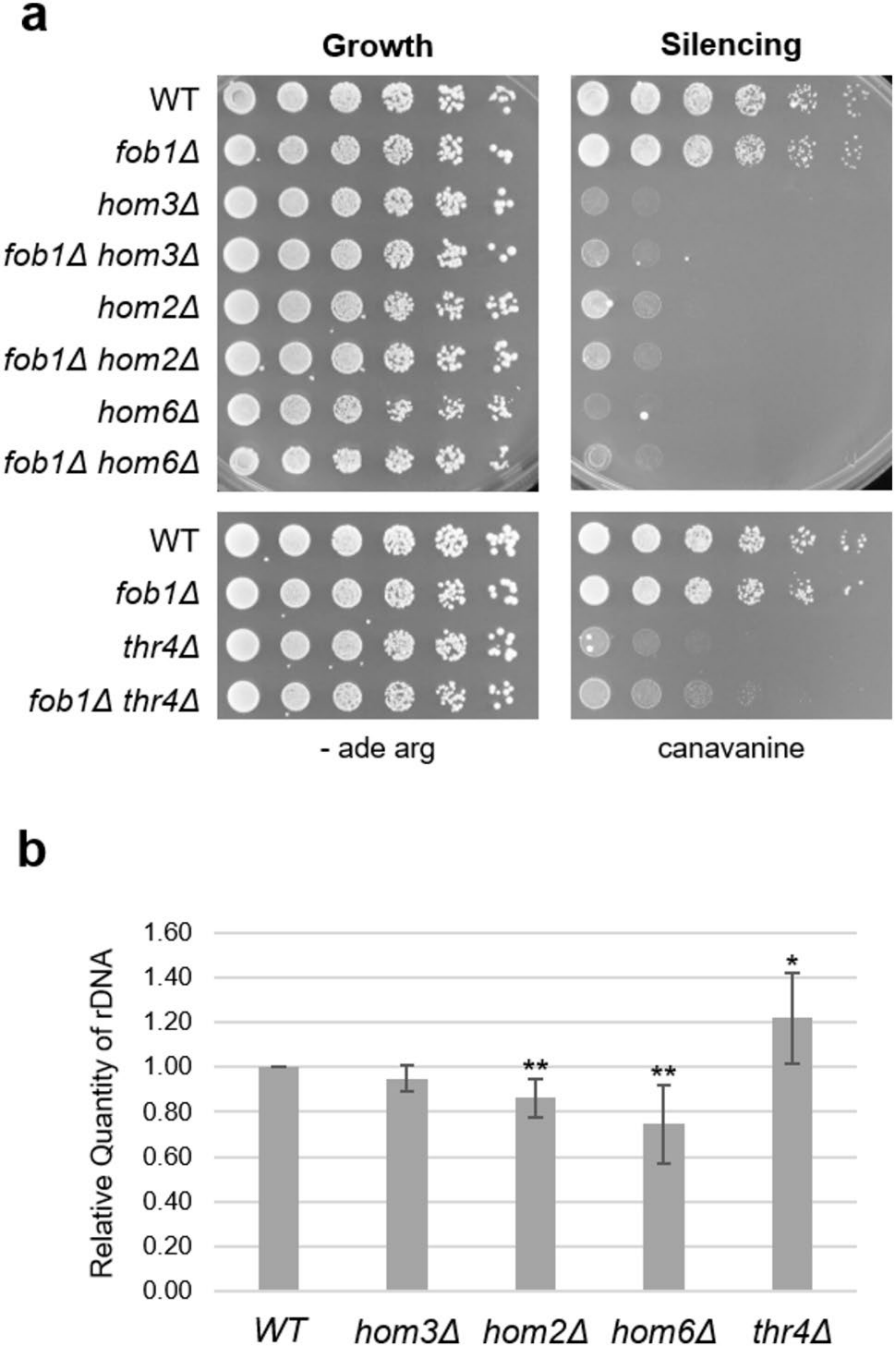
The threonine biosynthetic pathway does not promote rDNA silencing through alterations in rDNA copy number. (**a**) The *fob1Δ hom3Δ, fob1Δ hom2Δ, fob1Δ hom6Δ,* and *fob1Δ thr4Δ* double mutants have the same silencing phenotypes as their threonine pathway single mutant counterparts on 8 µg/mL canavanine. Imaged after a 3-day incubation. Strains shown: WT (LPY23157), *fob1Δ* (LPY23380)*, hom3Δ* (LPY16907)*, fob1Δ hom3Δ* (LPY23381)*, hom2Δ* (LPY16020)*, fob1Δ hom2Δ* (LPY23384)*, hom6Δ* (LPY23065)*, fob1Δ hom6Δ* (LPY23382)*, thr4Δ* (LPY23341)*, fob1Δ thr4Δ* (LPY23386). (**b**) qPCR analysis from two independent experiments reveals that *hom2Δ* (p = 0.0024)*, hom6Δ* (p = 0.0059), and *thr4Δ* (p = 0.0230) exhibit modest but statistically significant changes in rDNA copy number compared to WT. Students t-test was performed to determine significance relative to WT samples. Mean values ± SD *, p ≤ 0.05; **, p ≤ 0.01. Strains: WT (LPY23157, n = 6), *hom3Δ* (LPY16907, n = 5), *hom2Δ* (LPY16020, n = 6), *hom6Δ* (LPY23065, n = 6) *thr4Δ* (LPY23341, n = 6).

We took two approaches to address whether differences in copy number were a significant factor underlying the rDNA silencing defects we observed. In the first, we built on the observation that *FOB1*, a well-characterized regulator of rDNA copy number fluctuations^24^ also influences processes like silencing and replicative lifespan^25–27^. Notably, deletion of *FOB1* was shown to attenuate rDNA copy number effects over a large range of copy number differences^22^. We constructed *fob1Δ* double mutants and assayed *fob1Δ hom3Δ, fob1Δ hom2Δ, fob1Δ hom6Δ,* and *fob1Δ thr4Δ* strains for silencing. These double mutant strains had the same defective profiles as their single mutant counterparts with no exacerbation or suppression observed (Fig. 3a). In the second approach, we utilized a quantitative PCR (qPCR) assay^28^ that had been developed and validated to evaluate rDNA copy number. We observed modest alterations in *hom2Δ, hom6Δ*, and *thr4Δ* copy number when compared to WT strains (Fig. 3b). These molecular data, taken together with the *fob1Δ* functional data, suggest that rDNA copy number differences are unlikely to be major contributors to the means by which the threonine pathway mediates rDNA silencing.

Instead, we found that threonine itself could play a role in rDNA silencing in yeast as supplementation of media with excess threonine rescues rDNA silencing phenotypes in *hom3Δ, hom2Δ,* and *thr4Δ* strains (Fig. 2b). Supplementation with methionine did not rescue rDNA silencing phenotypes and supplementation with both amino acids in combination does not confer any additional growth advantage on medium containing canavanine (SFig. S1). This rescue is specific to the rDNA, as silencing defects at the telomeres are not affected by threonine supplementation (SFig. S2a). Rescue of rDNA silencing phenotypes is also unique to the threonine biosynthetic pathway as the phenotypes of other genes required for rDNA silencing, *ada2Δ, sir2Δ,* and various hypomorphic *sir2* alleles, were not rescued by excess threonine (Fig. 2b, SFig. S2b)^29–31^.

**Fig. 4:**
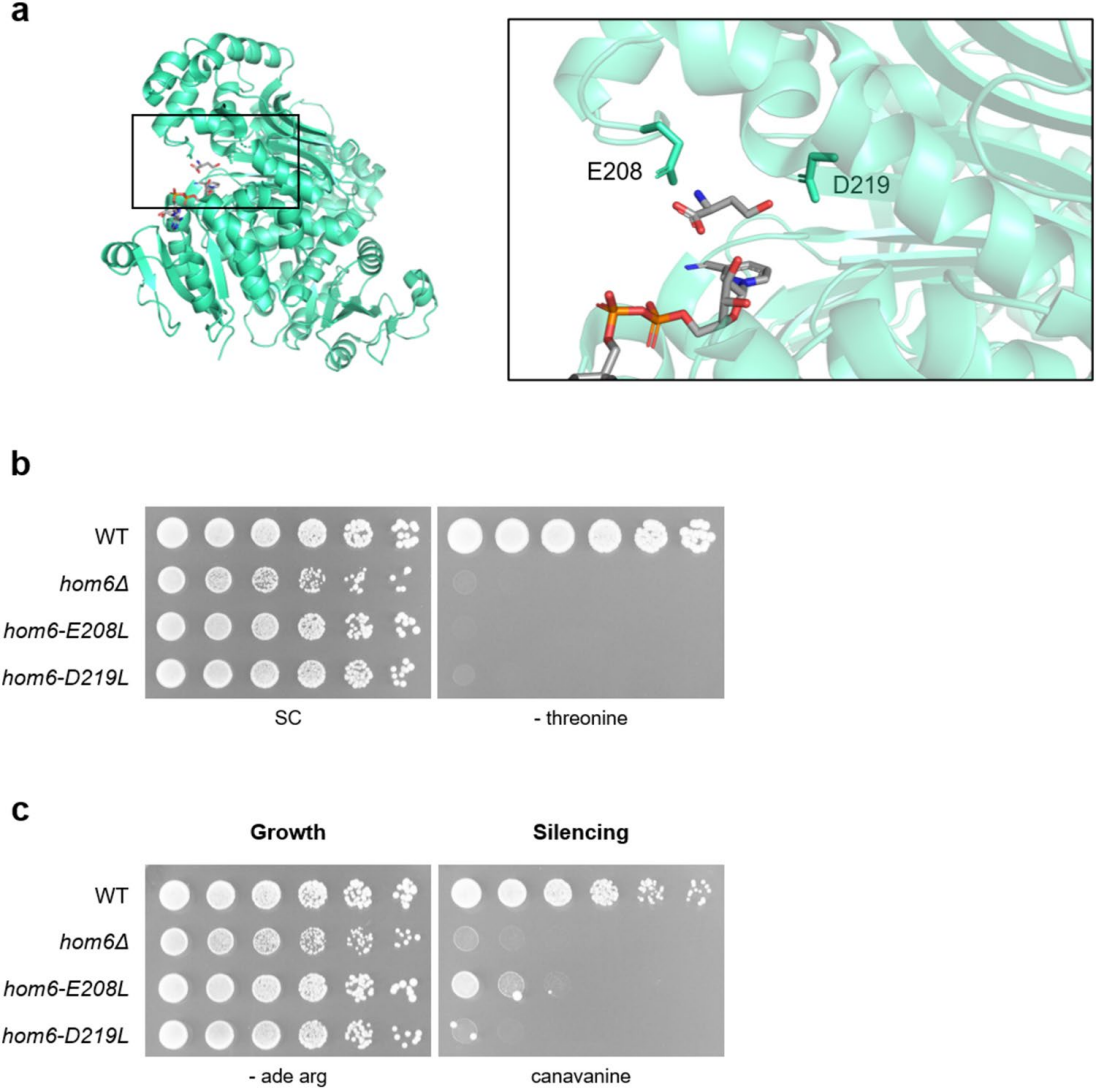
Hom6 catalytic mutants reveal distinct roles in rDNA silencing. (a) Hom6 structure and an expanded view of the Hom6 active site. E208 and D219 (green sticks), key residues for Hom6’s metabolic functions, were mutated to generate *hom6* catalytic mutants. Homoserine and 3-aminopyridine adenine dinucleotide (an NAD + analog) are shown in grey. PDB code: 1EBU. (**b**) CRISPR-generated mutants are defective for threonine biosynthesis. (**c**) The *hom6-E208L* strain retains partial silencing activity on 8 µg/mL canavanine. Imaged after 3 days. Strains presented: WT (LPY23157), *hom6Δ* (LPY23065), *hom6-E208L* (LPY23250), *hom6-D219L* (LPY23274).

Exogenous threonine did not rescue the rDNA silencing defects of *hom6Δ*, suggesting that Hom6 may regulate rDNA silencing through other previously uncharacterized activities. Hom6 has been thoroughly analyzed both structurally and kinetically^17^. The active site of Hom6 features a unique combination of polar residues that facilitate the conversion of aspartate semialdehyde to homoserine. Two residues, E208 and D219, are required for substrate binding and catalysis, respectively (Fig. 4a). To determine whether Hom6’s catalytic role in homoserine synthesis was required for its function at the rDNA, we utilized CRISPR-based mutagenesis to introduce the E208L and D219L mutations, into yeast cells^17^. Successful mutagenesis was verified with both Sanger sequencing and confirmation of threonine auxotrophy (Fig. 4b). Notably, when plated on canavanine, the two active site mutants exhibited differing phenotypes. Similar to *hom6Δ*, *hom6-D219L* is completely defective for rDNA silencing. However, *hom6-E208L* retained partial silencing activity (Fig. 4c). Thus, although both mutants are equally defective for threonine biosynthesis, *hom6-E208L* defines a moonlighting allele in which metabolic and chromatin-based activities are functionally distinguished.

### Hom6 is a positive regulator of NHEJ and coordinates with the MRX complex to mediate DNA damage repair

**Fig. 5:**
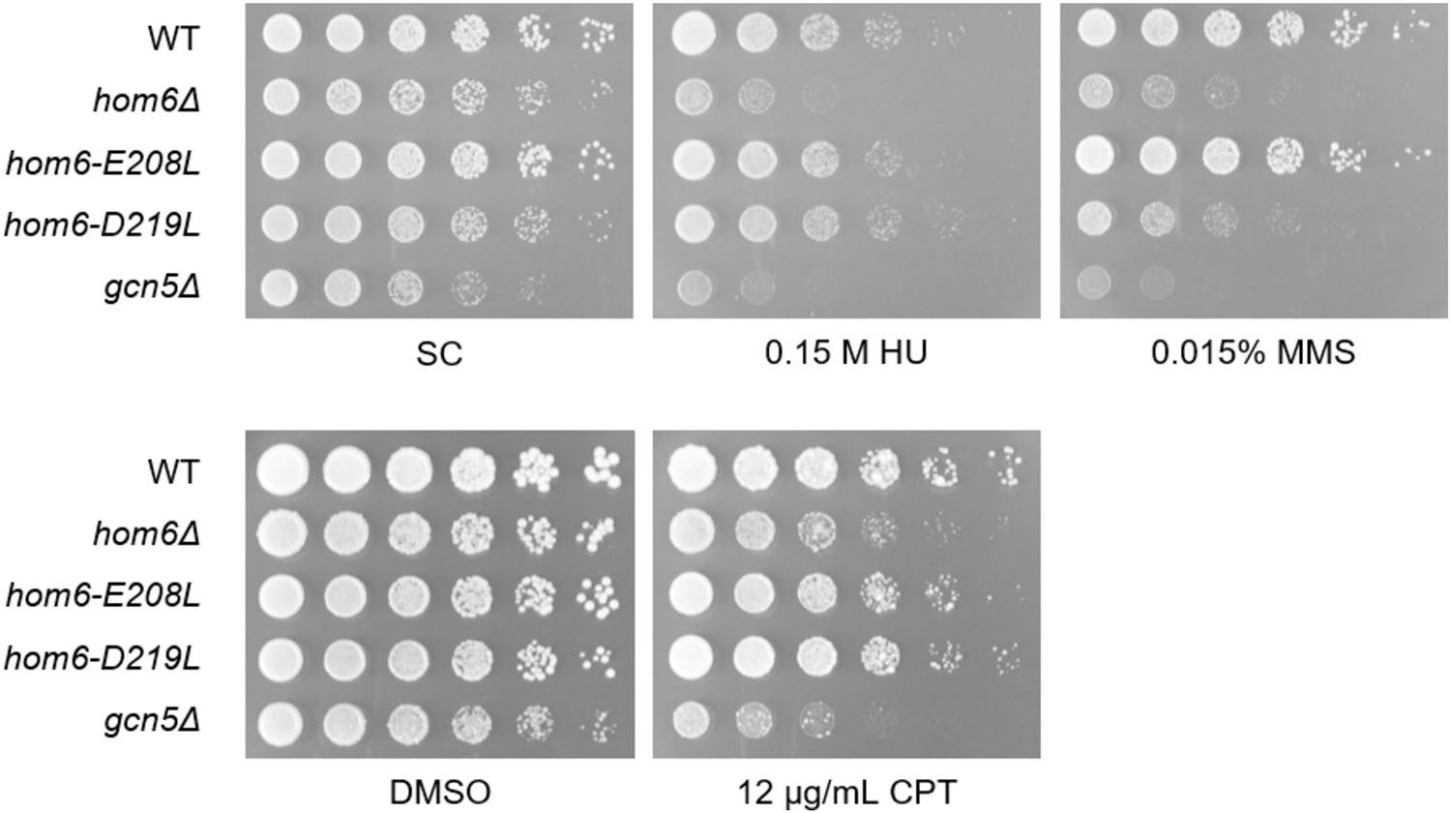
Hom6 is required for the DNA damage response. The *hom6Δ* mutant has increased sensitivity to 0.15 M HU, 0.015% MMS, and 12 µg/mL CPT. Catalytic mutants, *hom6-E208L* and *hom6-D219L*, have varying and distinct phenotypes in response to DNA damage challenges. HU and MMS plates are SC-based, whereas CPT plates contain buffered-YPAD. Growth control images were taken after a 2-day incubation. DNA damage plates were imaged after a 3-day incubation. Strains: WT (LPY23157), *hom6Δ* (LPY23065), *hom6-E208L* (LPY23250), *hom6-D219L* (LPY23274), *gcn5Δ* (LPY13435).

Chromatin-based functions are diverse and not limited to chromatin silencing or other aspects of transcriptional regulation. Thus, we also probed for roles of Hom6 in DNA repair. This characterization revealed that *hom6Δ* strains are sensitive to a variety of DNA damaging agents including the ribonucleotide reductase inhibitor hydroxyurea (HU), the topoisomerase inhibitor camptothecin (CPT), and the alkylating agent methane methylsulfonate (MMS) (Fig. 5). As in rDNA silencing, *hom6* catalytic mutants display distinct phenotypes when exposed to DNA damage challenges. Both mutants exhibit increased resistance to 0.15 M hydroxyurea and 12 µg/mL CPT when compared to *hom6Δ*. However, the *hom6-E208L* mutant uniquely shows resistance to 0.015% MMS (Fig. 5).

**Fig. 6:**
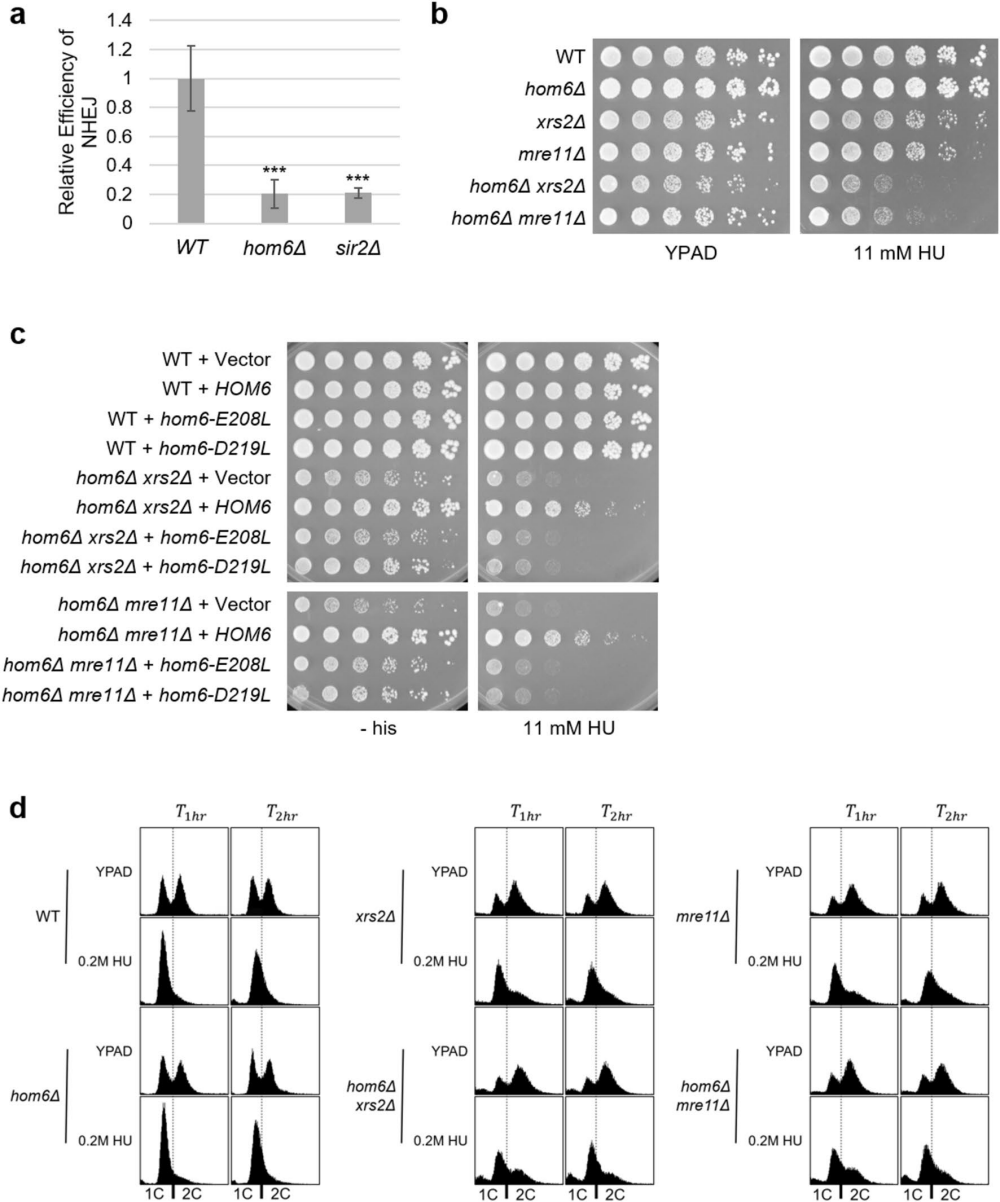
Hom6 is required for responses to DNA DSB repair. (**a**) *hom6Δ* strains are defective for NHEJ (p = 0.0002). *sir2Δ* serves as a positive control (p = 0.0006). Data are shown as the relative plasmid-end joining competency compared to WT. Students t-test was performed to determine significance relative to WT samples. Mean values ± SD. ***, p ≤ 0.001. Strains: WT (LPY6495, n = 5), *hom6Δ* (LPY23292, n = 6), and *sir2Δ (*LPY23320, n = 4). (**b**) The *hom6Δ xrs2Δ* and *hom6Δ mre11Δ* double mutant strains have heightened sensitivity to HU compared to either of their single mutant counterparts. Plates imaged after 3 days. Strains: WT (LPY6495), *hom6Δ* (LPY23292), *xrs2Δ* (LPY23282), *mre11Δ* (LPY23285), *hom6Δ xrs2Δ* (LPY23297), *hom6Δ mre11Δ* (LPY23296). (**c**) Hom6 catalytic activity is required to rescue *hom6Δ xrs2Δ* and *hom6Δ mre11Δ* DNA damage phenotypes. Cells were transformed with vector (pLP60) or a plasmid expressing *HOM6* (pLP2794)*, hom6-E208L* (pLP3542)*,* or *hom6-D219L* (pLP3546). DNA damage plates imaged after 4 days. Strains: WT (LPY6495), *hom6Δ xrs2Δ* (LPY23297), *hom6Δ mre11Δ* (LPY23296). (**d**) *hom6Δ xrs2Δ* and *hom6Δ mre11Δ* strains do not block the cell cycle as efficiently as WT and single mutant strains in response to HU treatment. More cells are found dead in a sub G1-peak and fail to respond to the HU-induced block in the double mutants.

Because hydroxyurea and camptothecin are both known to introduce DNA double strand breaks, we tested the competence of *hom6Δ* strains for NHEJ by performing plasmid end-joining assays^32,33^. In this assay, a supercoiled plasmid is transformed into yeast and a parallel transformation is performed with an enzymatically digested, linearized plasmid. Because the digested plasmid does not contain sequences homologous to the yeast genome at the site of the break, it must be repaired via the NHEJ mechanisms for propagation and subsequent cellular viability on selective media. Resulting transformant colony ratios of digested and supercoiled plasmid are indicative of the strain’s ability to perform NHEJ. We find that *hom6Δ* strains exhibit strong defects in NHEJ repair (Fig. 6a).

In considering mechanisms for sensitivity to DNA damage, we hypothesized that Hom6 might functionally interact with the MRX complex to mediate DSB repair, as high-throughput studies have previously categorized *HOM6* to be a negative genetic interactor with all components of the complex^34,35^. We chose to focus on two components of the MRX complex to better examine its relationship with Hom6. Mre11, a highly conserved nuclease, is responsible for DNA end processing^36^. Xrs2 binds to Mre11 and translocates the complex to the nucleus^37^. Double deletion strains *hom6Δ mre11Δ* and *hom6Δ xrs2Δ* were generated and subjected to DNA damage challenges. We observed that hydroxyurea sensitivity is greatly exacerbated in the double mutants compared to the respective single mutant strains (Fig. 6b). To determine whether Hom6’s catalytic activity is required for its relationship with the MRX complex, *hom6- E208L* and *hom6-D219L* plasmids were transformed into *hom6Δ mre11Δ* and *hom6Δ xrs2Δ* and plated on HU. The strains expressing the catalytically dead *hom6* mutants were as sensitive as their double deletion strain counterparts, indicating that Hom6’s catalytic activity is required for its functional relationship with the MRX complex (Fig. 6c).

To gain insight into the cellular response to DNA damage in the *hom6Δ mre11Δ* and *hom6Δ xrs2Δ* double mutants, we compared the cell cycle progression profiles of HU treated and untreated cells by flow cytometry. In HU, WT and *hom6Δ* cells exhibit a characteristic, tight S-phase arrest due to the reduction of ribonucleotide pools required for DNA synthesis. In contrast, cells without a functioning MRX complex reveal a defective S-phase checkpoint upon replicative stress and fail to arrest properly^38^. In comparison to WT and their single mutant counterparts when treated with HU, both *hom6Δ mre11Δ and hom6Δ xrs2Δ* display a broader peak containing a larger population of G2/M cells and a population of sub-G1 dead cells suggesting an even greater impairment of the S-phase checkpoint (Fig. 6d). Hence, Hom6 works in concert with the MRX complex to activate the S-phase checkpoint in response to DNA damage.

## Discussion

**Fig. 7:**
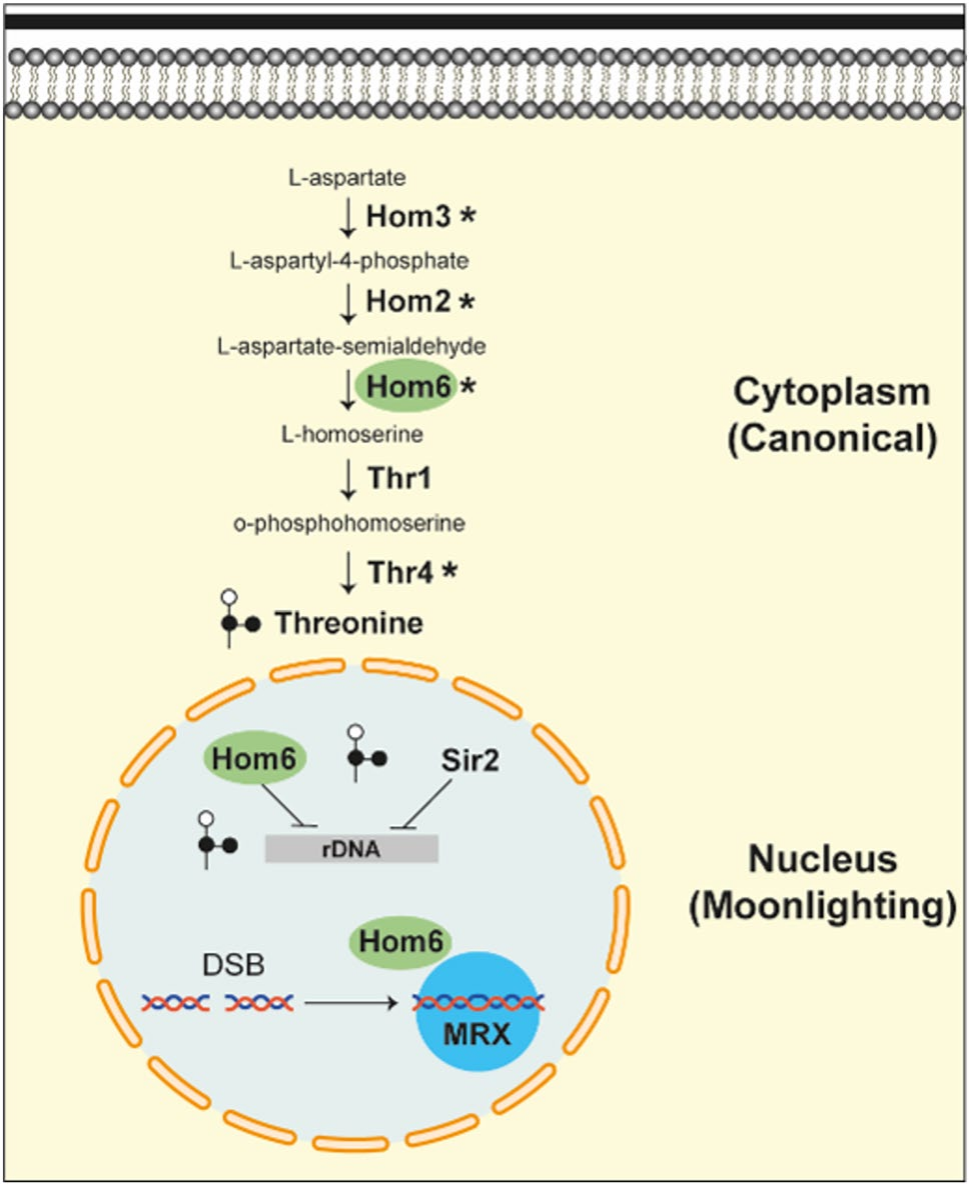
Hom6 contributes to canonical cytoplasmic functions and to a number of diverse nuclear roles. In the budding yeast *Saccharomyces cerevisiae*, a series of Hom and Thr proteins have well-established cytoplasmic roles in threonine synthesis. In addition to their cytoplasmic functions, the candidates chosen for this study(*) were found to promote and maintain silencing at the rDNA. Further investigation of Hom6's nuclear roles revealed its significance in DNA repair. Along with the MRX complex, Hom6 works to repair DNA DSBs via non-homologous end joining. Although we have no evidence for a direct physical interaction, our data reveal that Hom6 catalytic activity is required for this functional relationship.

Here, we show newly-discovered and diverse chromatin-based functions for enzymes within the yeast threonine biosynthetic pathway and specifically, a role for homoserine dehydrogenase in the regulation of DNA DSB repair (Fig. 7).

We demonstrate that the threonine biosynthetic pathway contributes to normal transcriptional silencing at the rDNA. This regulatory activity is scarcely altered by changes in Sir2 levels, a major player and often limiting factor in rDNA silencing. Although multiple genes in the threonine pathway contribute to silencing, their mechanisms of action are functionally distinct as *hom3Δ, hom2Δ*, and *thr4Δ* silencing defects are uniquely rescued by exogenous threonine. In the case of Hom3 and Hom2, silencing may be mediated by a mechanism that relies on other metabolites within the pathway, as Hom3 is not known to localize to the nucleus in *S. cerevisiae*^18^. Because Hom3 is feedback inhibited by threonine^39^, a *hom2Δ* strain supplemented with excess threonine will phenotypically mirror a *hom2Δ hom3Δ strain*. Therefore, candidate metabolites with roles in rDNA regulation include derivatives of both threonine and aspartate. Aspartate is interconnected with multiple metabolic pathways including the TCA cycle and nucleotide synthesis. In contrast to budding yeast, the *Schizosaccharomyces pombe* orthologs of Hom3, Hom2, and Hom6, are reported to be associated with heterochromatin and may thus mediate silencing directly^40^.

Although we observed modest differences in rDNA copy number among the mutants, this is unlikely to fundamentally influence the mechanism by which the threonine biosynthetic pathway promotes rDNA silencing. By qPCR, *hom2Δ, hom6Δ,* and *thr4Δ* strains display altered rDNA copy number in comparison to WT strains. There are limitations to this approach, because it may detect both genomic rDNA repeats and those which may have recombined as extra chromosomal circles. It is worth noting that previous high throughput studies utilized pulsed-field gel electrophoresis to analyze rDNA copy number and stability and did not report distinguishable differences between the threonine biosynthetic pathway deletion strains and WT^41^. As cellular phenotypes are known to vary depending on strain background, further investigation of the rDNA copy number is warranted. Even so, the unaltered silencing phenotypes of *fob1Δ* double mutant strains indicate that rDNA copy number is not likely to be a major contributing factor in threonine biosynthetic pathway-mediated rDNA silencing. These data agree with previous work in which strains with low rDNA copy number maintained normal silencing at the rDNA^21^.

When expanding the exploration of Hom6’s chromatin-based roles, we also found that Hom6 is required for responses to a variety of DNA damaging agents which trigger diverse mechanisms of repair. Hom6 is required for NHEJ competence and works with the MRX complex to repair DSBs. Strikingly, *hom6Δ mre11Δ* and *hom6Δ xrs2Δ* strains were hypersensitive to HU treatment. The defective S-phase checkpoint was exacerbated in the double mutants, therefore raising the possibility that these cells, already susceptible to DNA damage, accumulate additional damage as they are pushed through the checkpoint ultimately resulting in cell death. Taken together, these data indicate that Hom6 is required for activating NHEJ and the DNA damage checkpoint, thus coordinating with the MRX complex to repair damaged DNA. The MRX complex has well-characterized roles beyond DNA repair, including the maintenance of telomeric stability^42,43^. In our initial characterization, Hom6 was also found to have roles in promoting telomeric silencing^7^. Additional studies focusing on the functional relationship between Hom6 and MRX at other chromosomal loci would shed further insight on Hom6’s moonlighting functions in the nucleus.

Hom6’s contributions to chromatin biology are likely to be multi-faceted, with varying catalytic requirements for the maintenance of rDNA silencing, the DNA damage response, and functional interactions with the MRX complex. Both mutant strains, *hom6-E208L* and *hom6-D219L*, are auxotrophic for threonine, yet exhibit distinct phenotypes, demonstrating the existence of moonlighting alleles, in which catalytic and chromatin functions could be separated. Based on responses to DNA damaging agents, *hom6-E208L* is a traditional moonlighting allele in that Hom6’s amino acid metabolic function is independent of its functions in DNA repair. Curiously, *hom6-E208L* also retains partial silencing at the rDNA, indicating that Hom6’s role at the rDNA likely has multiple contributing factors including a limited dependency on its metabolic function. In contrast, *hom6-D219L* is fully defective in rDNA silencing, comparably to *hom6Δ.* However, the *hom6-D219L* strain shows increased resistance to HU and CPT treatment compared to the deletion strain, demonstrating the site’s importance for DSB repair. Neither *hom6* allele rescued *hom6Δ mrxΔ* phenotypes on HU indicating the absolute requirement for Hom6 catalytic activity. Overall, there is a degree of functional separation between Hom6’s role in DNA repair and its relationship with the MRX complex due to their differing requirements for Hom6 catalytic activity. Although neither mutant supports threonine synthesis, the two alleles vary kinetically and exhibit differing capacities for substrate and cofactor binding^17^. Independent evidence linking rDNA regulation and DNA repair comes from studies in which low rDNA copy number, often a result of genomic instability, has been shown to result in heightened sensitivity to DNA damage^23^. In addition, the MRX complex has also been reported to repair DSBs caused by replication fork barriers at the rDNA via a mechanism independent of traditional homology-based repair^44^. Further investigation of Hom6’s newly defined chromatin-based roles will reveal if their functional mechanisms intersect and will shed light on the far-reaching implications of Hom6 biology beyond amino acid metabolism.

An in silico screen first pointed to threonine biosynthetic enzymes as having potential roles in chromatin functions^7^. The current studies support that possibility, with a focus on rDNA silencing, and additional specific roles for *HOM6* and a moonlighting allele in DNA damage repair, mediated through the conserved MRX complex (Fig. 7). The results underscore the significance of metabolic enzymes in the evolution of chromatin and epigenetic processes^45,46^. The continued investigation of the moonlighting roles of non-mammalian, fungal enzymes, such as homocitrate synthase and the threonine metabolic enzymes presented here, expands the significance and motivation for their development as potential drug targets for the treatment of fungal diseases, particularly in the face of increasing populations of immunocompromised patients. Further exploration of all classes of moonlighting proteins will continue to enrich understanding of the evolution of complex biological functions.

## Methods

### Yeast strains and plasmids

Strains, plasmids, and oligonucleotides are listed in Table S1, S2, and S3, respectively. Null mutants were generated using standard molecular methods. Strains featured in Figs. 2, 3, 4 and 5 are of the W303 background. Strains in Fig. 6 are derived from the BY background and BY single deletion strains were obtained from the Yeast Deletion Collection^47^. The BY strains were used in the assays bridging DNA damage with the MRX complex because their phenotypes were consistent with the reported genetic interaction studies. In crosses and backcrosses, *hom6Δ* and *thr4Δ* strains were generated using a covering plasmid (pLP2628, pLP3075, or pLP3515).

### Growth assays

Cells were grown in YPAD, synthetic complete (SC), or defined drop-out media at 30 °C, for 1–2 days and normalized to an A_600_ of 1.0. Five-fold serial dilutions were plated and photographed for 2–5 days after plating. To assay for rDNA silencing, cells were plated on SC-ade-arg and SC-ade-arg with canavanine ranging from 8 to 40 µg/mL. Cells were plated on SC and SC with 0.5 mg/mL 5-FOA to monitor telomeric silencing. For the amino acid supplementation experiments, threonine and methionine solutions were added to plates resulting in final concentrations of 0.6 mg/mL and 0.06 mg/mL, respectively. Drug concentrations are noted in figure legends. Camptothecin plates were YPAD-based and buffered to pH 7.5 with 100 mM potassium phosphate^48^. Growth control plates were also buffered and contained the equivalent volume of the DMSO solvent for camptothecin. MMS plates were SC-based. HU plates were SC, YPAD, and SC-his -based in Figs. 5, 6b, and c, respectively.

### qPCR

Genomic DNA was prepared from liquid cultures by standard phenol chloroform extraction methods followed by RNase A treatment. qPCR reactions targeting *BUD23* and 25S rDNA were then performed with SsoAdvanced Universal SYBR Green Supermix (Bio-Rad) using primers and cycling conditions as previously reported^28^.

### CRISPR-mediated mutagenesis

*hom6-E208L* and *hom6-D219L* catalytic mutant strains were generated via CRISPR-based mutagenesis. CRISPR Direct (https://crispr.dbcls.jp/) was used to design a gRNA targeting the *HOM6* locus^49^. The gRNA oligonucleotides, containing a 5’ BclI overhang and 3’ partial sgRNA sequence, were subsequently hybridized and ligated into the BclI and SwaI sites of pML104^50^ (a generous gift from L. McDonnell, UCSD) producing pLP3510. The homology directed repair (HDR) template was synthesized via PCR from two overlapping oligonucleotides containing the mutations of interest and a silent mutation to disrupt the PAM sequence. Cells were transformed with 250–500 ng of pLP3510 and 2 µg of HDR template with lithium acetate methods. The *hom6* coding region was amplified via PCR from threonine auxotrophic candidates and the presence of the mutations was confirmed via Sanger sequencing. Of note, the *hom6-D219L* mutant retained its native PAM sequence. Oligonucleotides used for mutant generation and sequencing are listed in Table S3.

### Plasmid end-joining assays

Assays using pLP60 (pRS313) and SacI-HF digested DNA were performed with established methods^32,33^. Samples from three independent experiments were used for quantification. Student’s t-test was used to determine significance.

### Flow cytometry

Assays and analysis were performed as previously described^51^. HU was added to log-phase cultures to a final concentration of 0.2 M and samples were collected for processing at one and two hours after treatment.

## Supplementary Information

Supplementary Information.

## Data Availability

Raw qPCR data, yeast strains, and plasmids will be provided upon request.
