# Supplementary material for: Non-canonical chromatin-based functions for the threonine metabolic pathway
Source: Sci Rep. 2024 Sep 30;14:22629. doi: 10.1038/s41598-024-72394-z (PMC11442984; doi:10.1038/s41598-024-72394-z)
Supplement: Supplementary file 1 — Supplementary Information. [file 41598_2024_72394_MOESM1_ESM.pdf]

Supplementary Information

Non-canonical chromatin-based functions for the threonine metabolic pathway

Jennifer K. Chik[1], Xue Bessie Su[1,2], Stephen Klepin[1], Jessica Raygoza[1], and Lorraine Pillus[1*]

[1]Department of Molecular Biology, University of California San Diego, 9500 Gilman Drive, La Jolla, CA 92093-0347, USA.
[2]Medical Research Council, Laboratory for Molecular Cell Biology, University College London, London, WC1E 6BT, UK
[*]lpillus@ucsd.edu

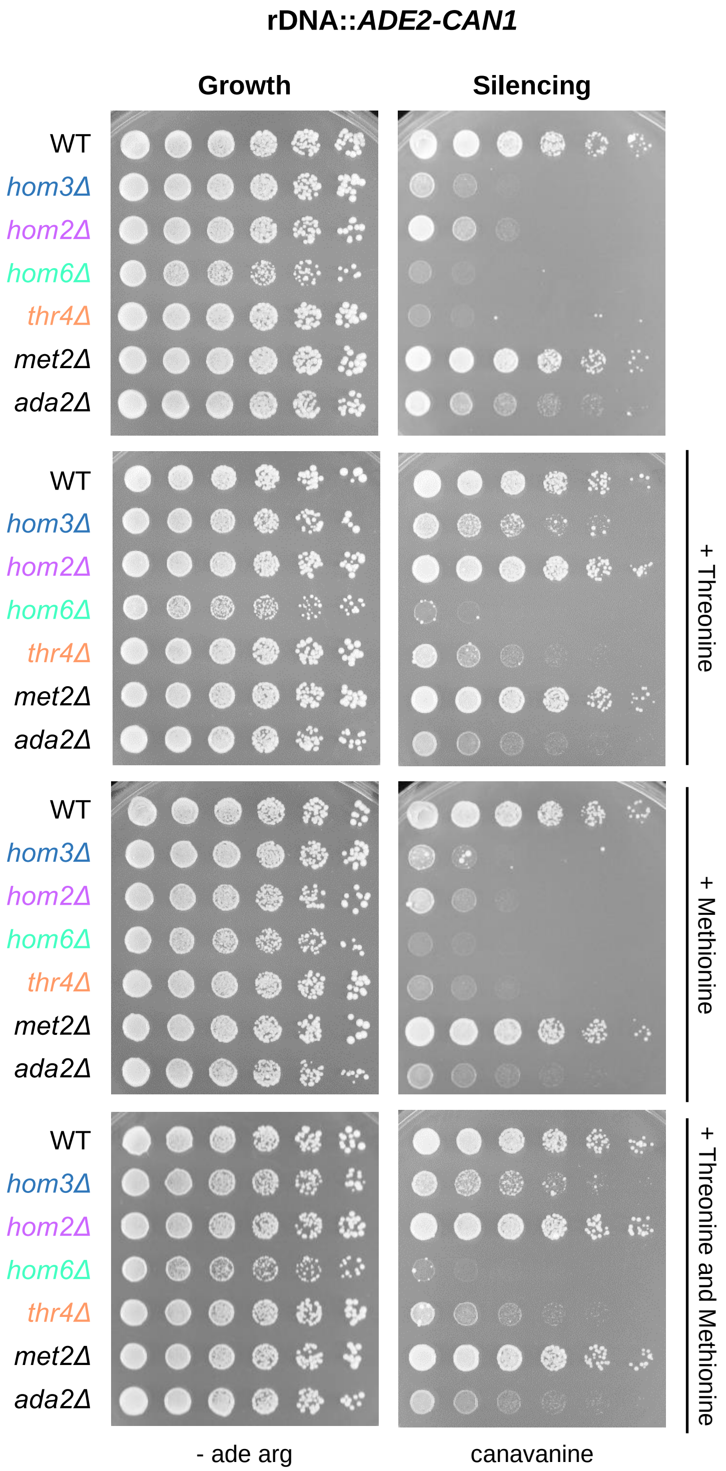

Figure S1. Methionine supplementation does not affect rDNA silencing.
SC -ade arg and SC -ade arg 8 µg/mL canavanine plates were top-coated with excess threonine, methionine, or a combination of threonine and methionine and assayed for defects in rDNA silencing. Methionine supplementation does not rescue the rDNA silencing defects of any strain, including *hom3Δ*, *hom2Δ,* and *thr4Δ.* Strains presented as in Figure 2. Imaged after a 3-day incubation.

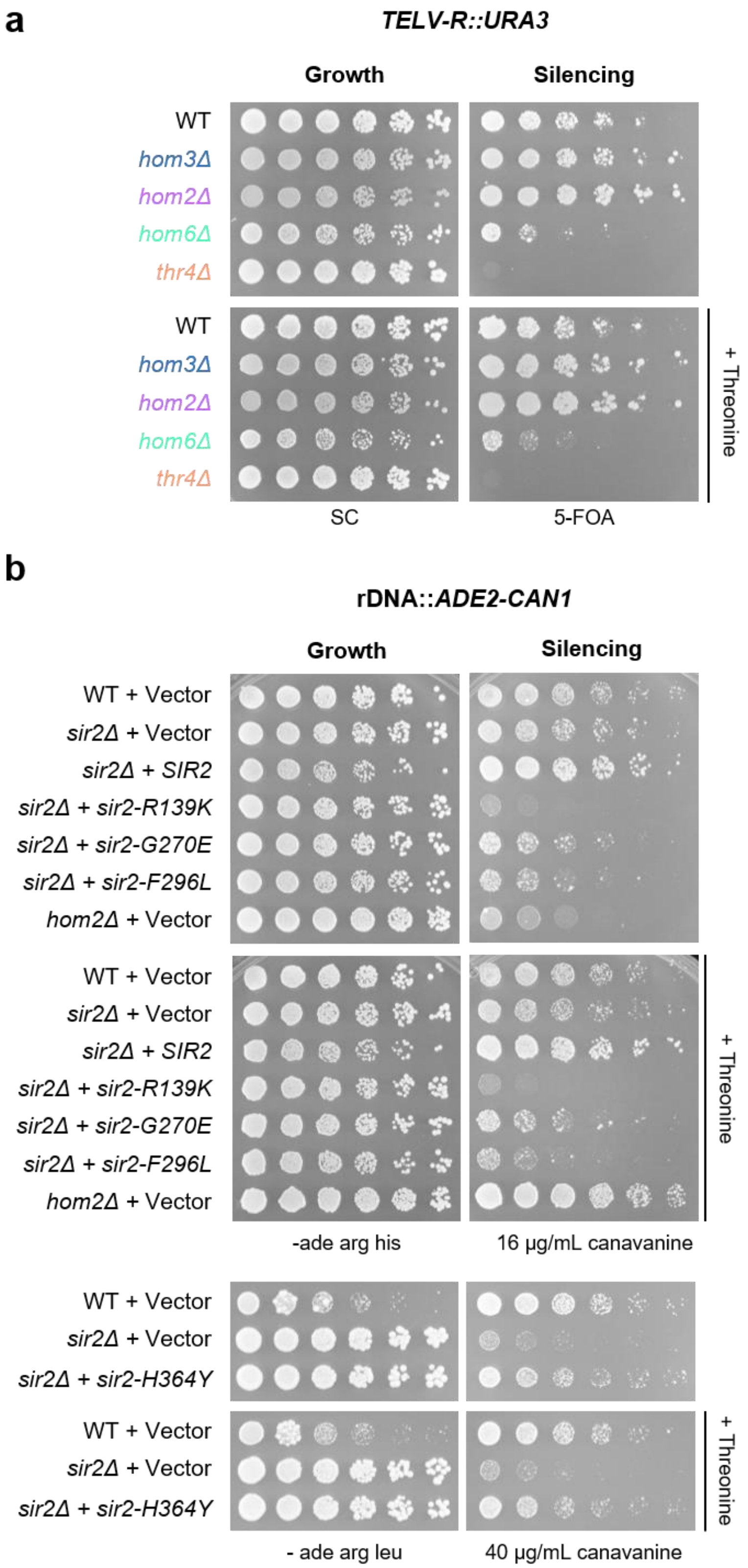

a
TELV-R::URA3
Growth
Silencing
WT
hom3Δ
hom2Δ
hom6Δ
thr4Δ
WT
hom3Δ
hom2Δ
hom6Δ
thr4Δ
+ Threonine
SC
5-FOA
b
rDNA::ADE2-CAN1
Growth
Silencing
WT + Vector
sir2Δ + Vector
sir2Δ + SIR2
sir2Δ + sir2-R139K
sir2Δ + sir2-G270E
sir2Δ + sir2-F296L
hom2Δ + Vector
WT + Vector
sir2Δ + Vector
sir2Δ + SIR2
sir2Δ + sir2-R139K
sir2Δ + sir2-G270E
sir2Δ + sir2-F296L
hom2Δ + Vector
+ Threonine
-ade arg his
16 μg/mL canavanine
WT + Vector
sir2Δ + Vector
sir2Δ + sir2-H364Y
WT + Vector
sir2Δ + Vector
sir2Δ + sir2-H364Y
+ Threonine
- ade arg leu
40 μg/mL canavanine

Figure S2. Threonine supplementation specifically rescues threonine biosynthetic pathway rDNA silencing phenotypes.

(a) Threonine supplementation does not improve defects in telomeric silencing. Strains contain a *URA3* reporter gene at the right arm of telomere V, where it is epigenetically silenced. Decreased growth on 5-FOA is indicative of a defect in *URA3* silencing. *hom6Δ* and *thr4Δ* exhibit defects in telomeric silencing that are not rescued by threonine supplementation. Images were taken after a 3-day incubation. Strains Plated: WT (LPY23157), *hom3Δ* (LPY16903), *hom2Δ (*LPY16034), *hom6Δ* (LPY23065), *thr4Δ* (LPY23341).

(b) Supplementation with threonine does not rescue rDNA silencing defects of *sir2* hypomorphic alleles. Cells were transformed with either vector (pLP 60 or pLP400), a *SIR2* plasmid (pLP285), or a plasmid with mutant alleles of *SIR2* (pLP1102, pLP1110, pLP1112, pLP1976). Imaged after a 3-day incubation. Strains: WT (LPY23157), sir2Δ (LPY5013), *hom2Δ* (LPY16020).

Table S1. Yeast Strains

Except where noted, strains were constructed during the course of this study. Strains LPY5013-LPY23386 are of the W303 background and LPY6495-LPY23320 are of the BY background.

Strain	Genotype
LPY5013	MATα W303 sir2Δ::TRP1 rDNA::ADE2-CAN1
LPY11674	MATα W303 ada2Δ::kanMX rDNA::ADE2-CAN1
LPY13435	MATα W303 gcn5Δ::natMX
LPY16020	MATα W303 hom2Δ::kanMX rDNA::ADE2-CAN1
LPY16034	MATα W303 hom2Δ::kanMX TELV-R::URA3
LPY16903	MATα W303 hom3Δ::kanMX TELV-R::URA3
LPY16907	MATα W303 hom3Δ::kanMX rDNA::ADE2-CAN1
LPY23065	MATα W303 hom6Δ::kanMX hmrΔE::TRP1 rDNA::ADE2-CAN1 TELV-R::URA3
LPY23157	MATα W303 hmrΔE::TRP1 rDNA::ADE2-CAN1 TELV-R::URA3
LPY23250	MATα W303 hom6-E208L hmrΔE::TRP1 rDNA::ADE2-CAN1
LPY23274	MATα W303 hom6-D219L hmrΔE::TRP1 rDNA::ADE2-CAN1
LPY23330	MATα W303 met2Δ::kanMX hmrΔE::TRP1 rDNA::ADE2-CAN1 TELV-R::URA3
LPY23341	MATα W303 thr4Δ::kanMX hmrΔE::TRP1 rDNA::ADE2-CAN1 TELV-R::URA3
LPY23380	MATα W303 fob1Δ::kanMX hmrΔE::TRP1 rDNA::ADE2-CAN1 TELV-R::URA3
LPY23381	MATα W303 fob1Δ::kanMX hom3Δ::kanMX hmrΔE::TRP1 rDNA::ADE2-CAN1 TELV-R::URA3
LPY23382	MATα W303 fob1Δ::kanMX hom6Δ::kanMX hmrΔE::TRP1 rDNA::ADE2-CAN1 TELV-R::URA3
LPY23384	MATα W303 fob1Δ::kanMX hom2Δ::kanMX rDNA::ADE2-CAN1
LPY23386	MATα W303 fob1Δ::kanMX thr4Δ::kanMX hmrΔE::TRP1 rDNA::ADE2-CAN1 TELV-R::URA3
LPY6495	MATa his3Δ1 leu2Δ0 met15Δ0 ura3Δ0
LPY23282	MATa his3Δ1 leu2Δ0 met15Δ0 ura3Δ0 xrs2Δ::kanMX
LPY23285	MATa his3Δ1 leu2Δ0 met15Δ0 ura3Δ0 mre11Δ::kanMX

LPY23292	*MAT*a *his3Δ1 leu2Δ0 met15Δ0 ura3Δ0 hom6Δ::kanMX*
LPY23296	*MAT*a *his3Δ1 leu2Δ0 lys2Δ0 ura3Δ0 hom6Δ::kanMX mre11Δ::kanMX*
LPY23297	*MAT*a *his3Δ1 leu2Δ0 lys2Δ0 met15Δ0 ura3Δ0 hom6Δ::kanMX xrs2Δ::kanMX*
LPY23320	*MAT*a *his3Δ1 leu2Δ0 met15Δ0 ura3Δ0 sir2Δ::kanMX*

Table S2. Plasmids

Plasmid	Description	Marker
pLP60*	pRS313	*HIS3*
pLP270**	pRS423	*HIS3*
pLP285***	pRS313-*SIR2*	*HIS3*
pLP400*	pRS315	*LEU2*
pLP891	pRS423-*SIR2*	*HIS3*
pLP1102***	pRS313-*sir2-R139K*	*HIS3*
pLP1110***	pRS313-*sir2-G270E*	*HIS3*
pLP1112***	pRS313-*sir2-F296L*	*HIS3*
pLP1976****	pRS315-*sir2-H364Y*	*LEU2*
pLP2628	pRS315-*HOM6*	*LEU2*
pLP2794	*pRS313-HOM6*	*HIS3*
pLP3075	pRS316-*HOM6*	*URA3*
pLP3510	pML104-*HOM6* gRNA	*URA3*
pLP3515	pRS423-*THR4*	*HIS3*
pLP3542	pRS313-*hom6-E208L*	*HIS3*
pLP3546	pRS313-*hom6-D219L*	*HIS3*

* Sikorski, R. S. & Hieter, P. A system of shuttle vectors and yeast host strains designed for efficient manipulation of DNA in Saccharomyces cerevisiae. *Genetics* 122, 19-27 (1989).
****** Christianson, T. W., Sikorski, R. S., Dante, M., Shero, J. H. & Hieter, P. Multifunctional yeast high-copy-number shuttle vectors. *Gene* 110, 119-122 (1992).
*** Garcia, S. N. & Pillus, L. A unique class of conditional *sir2* mutants displays distinct silencing defects in *Saccharomyces cerevisiae. Genetics* 162, 721-736 (2002).
****Tanny, J. C., Dowd, G. J., Huang, J., Hilz, H. & Moazed, D. An enzymatic activity in the yeast Sir2 protein that is essential for gene silencing. *Cell* 99, 735-745 (1999).

Table S3. Oligonucleotides

Oligo Number	Name	Sequence (5’ → 3’)
oLP1516	HOM2_5_KO	CGACGGAGAAGAAGGAGAC
oLP1517	HOM2_3_KO	CTTGGGTCAGCGAGAGAATTAC
oLP1604	MET2_5KO	CAGCTGCGTCCAATAGATGAG
oLP1606	HOM3_5KO	CTTTCCGTACGCAGTCTTCTC
oLP1607	HOM3_3KO	GTCAGTGATGGGGATTTGC
oLP1769	hom6_5KOnew	CAATAACGCACATGGTGG
oLP1770	hom6_3KOnew	GCCCCATGACATGGATGAG
oLP2493	MET2_3KO_V2	CGGTAACTCGTGTGCTCTCATTC
oLP2496	THR4_5KO_V2	GTCTTCCAAGCAAAACGAGC
oLP2497	THR4_3KO_V2	GGTTCCTTACTCTCGCAATC
oLP2516	THR4SpeI_F	CGATACTAGTTGCATCCAAGAGAAACGCTG
oLP2517	THR4ClaI_R	GGCATCGTTAGTGTATGCACATCCTG
oLP2531	MRE11KO_JKC_F	GTCAGAGTTCACAAGCAAGCC
oLP2532	MRE11KO_JKC_R	CGAACAAAAGAGCAAAGGCTGG
oLP2533	XRS2KO_F	CGTAAAGTTGTTAACTACGC
oLP2534	XRS2KO_R	AGCAACCTGTAGTGCTTC
oLP2535	hom6_E208L_HDRF	GGGTTATACTTTACCAGATCCAAGAGATGATTTGAATGGG TTGG
oLP2536	hom6_E208L_HDRR	GAGATTCAACTTCCACACCAGATATCCTACCAACAATGGT AACTTTTCTAGCAACATCCAACCCATTCAAATCATC
oLP2537	hom6_D219L_HDRF_v2	GGGTTATACTGAACCAGATCCAAGAGATGATTTGAATGGG TTGTTGGTTGCTAGAAAAG
oLP2538	hom6_D219L_HDRR_v2	GAGATTCAACTTCCACACCAGATATCCTACCAACAATGGT AACTTTTCTAGCAACCAAC
oLP2541	HOM6-XhoI-F	AAAGCACTCGAGACCCTACTTC
oLP2542	HOM6-SacI-R	CTGCTTCGAGCTCTTCATTAG
oLP2547*	BUD23F-QPCR	GACCATGTGTGGTGTGGTTT

oLP2548*	BUD23R-QPCR	CCCATATCCTGCAACATCAA
oLP2549*	25SF-QPCR	GAATCCATATCCAGGTTCCG
oLP2550*	25SR-QPCR	GACGTGGGTTAGTCGATCCT
oLP2551	FOB1KO-F	GATCGAGGTTTCCAGGAAGAG
oLP2552	FOB1KO-R	CGGGCAAGATCATATTATCCAAGTTG
oLP2559	HOM6-intseqR	GATAGAGTACCAGAGAAGATACC

* Jack, C. V. et al. Regulation of ribosomal DNA amplification by the TOR pathway. *Proc Natl Acad Sci U S A* 112, 9674-9679 (2015).
